# Estimation of genetic relationships between growth curve parameters in Guilan sheep

**DOI:** 10.1186/s40781-015-0052-6

**Published:** 2015-05-14

**Authors:** Navid Ghavi Hossein-Zadeh

**Affiliations:** Department of Animal Science, Faculty of Agricultural Sciences, University of Guilan, P. O. Box: 41635–1314, Rasht, Iran

**Keywords:** Bayesian methodology, Fat-tailed sheep, Genetic parameters, Genetic trend, Growth curve

## Abstract

The objective of this study was to estimate variance components and genetic parameters for growth curve parameters in Guilan sheep. Studied traits were parameters of Brody growth model which included A (asymptotic mature weight), B (initial animal weight) and K (maturation rate). The data set and pedigree information used in this study were obtained from the Agricultural Organization of Guilan province (Rasht, Iran) and comprised 8647 growth curve records of lambs from birth to 240 days of age during 1994 to 2014. Marginal posterior distributions of parameters and variance components were estimated using TM program. The Gibbs sampler was run 300000 rounds and the first 60000 rounds were discarded as a burn-in period. Posterior mean estimates of direct heritabilities for A, B and K were 0.39, 0.23 and 0.039, respectively. Estimates of direct genetic correlation between growth curve parameters were 0.57, 0.03 and −0.01 between A-B, A-K and B-K, respectively. Estimates of direct genetic trends for A, B and K were positive and their corresponding values were 0.014 ± 0.003 (P < 0.001), 0.0012 ± 0.0009 (P > 0.05) and 0.000002 ± 0.0001 (P > 0.05), respectively. Residual correlations between growth curve parameters varied form −0.52 (between A-K) to 0.48 (between A-B). Also, phenotypic correlations between growth curve parameters varied form −0.49 (between A-K) to 0.47 (between A-B). The results of this study indicated that improvement of growth curve parameters of Guilan sheep seems feasible in selection programs. It is worthwhile to develop a selection strategy to obtain an appropriate shape of growth curve through changing genetically the parameters of growth model.

## Background

Growth is an economically important trait of farm animals which can be interpreted mathematically. It is a time (age) dependent change expressed in the weight or size of organ, composition of tissue/organ, size or number of cells and in live weight [1]. Growth rate is related to rate of maturing and mature weight and these traits have been suggested to have association with lifetime productivity parameters in animals [2]. Slow growth rate causes low market weight and has been identified as one of the limiting factors affecting the profitability of any production system [3].

Growth models have been used extensively in different species to describe the development of body weight, allowing information from multiple measurements to be combined into a few (usually 3 or 4) variables [4]. The fit of a growth function, and hence the variables estimated, will depend on the number and timing of available body weight observations [4]. Among a number of non-linear mathematical models, Brody function [5] has been found to be adequate for comparing individual differences in rate of maturing and mature weight. In addition, this model has advantages of mathematical simplicity and biological interpretability. Recently, other researchers have applied this model for fitting growth curve in sheep [3,6,7].

Accurate estimates of variance components are important in animal breeding because genetic parameters are dependent on (co) variance components [8]. Therefore, improved methodologies for estimation of these components are constantly evolving. The Gibbs Sampling algorithm [9] enables estimation of random samples of parameter estimates based on a given data set, by generating posterior distributions of parameter values that are proportional to the product of the parameter probabilities and the observation probabilities [10].

The ability to modify the shape of the growth curve may be an attractive aspect for livestock breeders (e.g., to increase early growth but restrict mature size, and hence maintenance requirements). To determine the genetic flexibility of the shape of growth curves, genetic parameters must be calculated for the underlying curve variables [4]. Estimates of genetic parameters and genetic trends for body weight traits in Guilan sheep were reported in a previous study [11], but estimates of variance components and heritabilities for growth curve parameters in sheep are very scarce in the literature. The objective of this study was to estimate variance components and genetic parameters for growth curve parameters in Guilan sheep using Gibbs sampling algorithm by Bayesian methodology. The results of the current study would indicate the possibility of implementing genetic selection for growth curve parameters in Guilan sheep. Also, current estimates of genetic parameters could be used for designing future selection schemes for Guilan breed, with the genetic improvement of growth rate being one of the main parts of selection goals.

## Methods

The Guilan sheep is a fat-tailed breed of domestic sheep in Iran, numbering some 400,000 animals in the north of the country, and distributed in the northern and western parts of Guilan Province. Mean adult live weight in this breed is 35 kg (77 lbs) for rams and 31 kg (67 lbs) for ewes. This breed is valued mainly due to its ability to live in mountainous areas with rain-fed foothills and foothill steppes. Young ewes were randomly exposed to the rams for the first time at approximately 1.5 years of age. Ewes were kept in the flock up to 7 years of age. Ewes are supplemented, depending upon the ewes’ requirements, for a few days after lambing. During the breeding season, single-sire pens were used allocating 20–25 ewes per ram. Lambs remained with their dam until weaning. Lambs were ear-tagged and weighted immediately after lambing. During the suckling period, lambs suckled their mothers while being allowed dry alfalfa after 3 weeks of age. Lambs are weaned at approximately 90 days of age. Animals are kept on natural pasture during spring, summer and autumn seasons. Since environmental conditions are not suitable during the winter, therefore the animals are kept indoors during the winter months. The flock was mainly kept on range and fed cereal pasture, but supplemental feed, including alfalfa and wheat straw, are provided especially around mating season [12].

The data set and pedigree information used in this study were obtained from the Agricultural Organization of Guilan province (Rasht, Iran) and comprised 8647 growth curve parameters of lambs from birth to 240 days of age during 1994 to 2014. Brody model was used to estimate parameters of the growth curve for each animal and is presented as follows:

$$ y=A\left(1-B{e}^{-Kt}\right) $$

Where: y represents body weight at age t (days); A represents asymptotic weight, which is interpreted as mature weight; and B is an integration constant related to initial animal weight. The value of B is defined by the initial values for y and t; and K is the maturation rate, which is interpreted as weight change in relation to mature weight to indicate how fast the animal approaches adult weight. The data were screened several times and atypical growth curves resulting in out of range parameters were deleted. Lambs with incomplete records of parentage or with registration numbers lower than the numbers of their parents were left out. Averages of A, B and K parameters were 29.37 ± 9.67 kg, 0.89 ± 0.05 kg/day and 0.0069 ± 0.0035 kg gain per kg live weight per day, respectively. The whole number of animals, with sires, dams, founders and non-founders included in the pedigree data set were 21736, 304, 7154, 10275 and 11461, respectively.

The selection of fixed effects to be included in the model was made after testing their statistical significance using the General Linear Model (GLM) procedure of SAS [13]. The level of significance for the inclusion of effects into the model of analysis was declared at P < 0.05. The final model for A parameter included the fixed effects of flock, birth year, sex, litter size and age of dam. The final model of analysis for B and K parameters included the same independent variables, together with birth month effect. All models of analysis included the random additive genetic effect of animal. The univariate animal model fitted for the genetic analysis of the growth curve parameters was as follows:

$$ \boldsymbol{y}=\boldsymbol{X}\boldsymbol{b}+\boldsymbol{Z}\boldsymbol{a}+\boldsymbol{e} $$

Where, **y** is a N × 1 vector of records for each growth curve parameter, **b** denotes the fixed effects in the model with association matrix **X**, **a** is the vector of direct genetic effects with the incidence matrix **Z** and **e** denotes the vector of residual effects. From a Bayesian perspective, it was assumed that prior distribution for additive direct effects had a multivariate normal distribution with mean 0 and variance **A***σ*_*a*_^2^, where **A** is the additive numerator relationship matrix and *σ*_*a*_^2^ is the additive direct variance. Residual effects were assumed to be multivariate normally distributed with mean 0 and variance **I**_**n**_*σ*_*e*_^2^, where **I**_**n**_ is identity matrix with order equal to the number of individual records, respectively and *σ*_*e*_^2^ is the residual variance. Bivariate or pair-wise analyses were carried out for every pair of traits. The models applied in pair-wise analyses were those fitted for each of the traits in the univariate analyses.

The prior distribution for the genetic and residual (co)variance matrices was assumed to be an inverted Wishart distribution. Marginal posterior distributions of parameters and variance components were estimated, using TM program [14]. The Gibbs sampler was run 300000 rounds, and the first 60000 rounds were discarded as a burn-in period [14]. A thinning interval of 100 rounds was used to retain sampled values that reduced lag correlation among thinned samples. The Bayesian Output Analysis (BOA version 1.1.5; [15]) package was used to calculate the mean, SD, and 95% high posterior density (HPD) interval for all parameters from the individual marginal posteriors, under the free software R (http://www.r-project.org/). The HPD region or confidence interval provides the interval that includes 95% of samples and is a measure of reliability. Also, the HPD can be applied to non-symmetric distributions [16]. Genetic trends were obtained by regressing yearly mean estimates of breeding values on birth year. Phenotypic trends of growth curve parameters were estimated using the linear regression of average phenotypic values on birth year.

## Results and discussion

**Figure 1:**
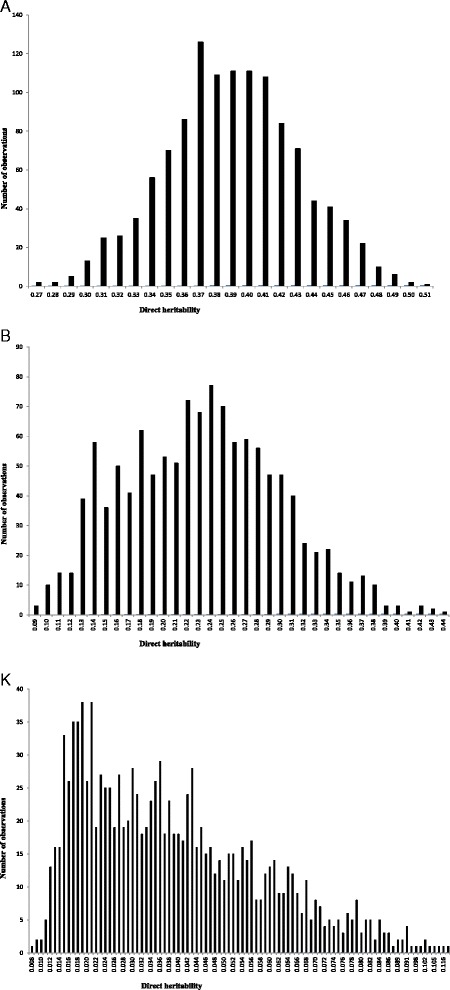
Posterior distribution of the direct heritabilities for **A**, **B** and **K** growth curve parameters.

The posterior means, medians and modes of variance components and direct heritabilities and their HPDs for growth curve parameters obtained from univariate models of analysis are presented in Table 1. Posterior mean estimates of direct heritabilities for A, B and K were 0.39, 0.23 and 0.039, respectively. Medium to high heritability estimates for A and B parameters imply that much of the improvement on these parameters could be attained by genetic selection. The low estimate of heritability for K implies that much of the improvement on this growth curve parameter could be obtained by improving associated environmental conditions rather than genetic selection [17,18]. It is important to note that the variation range of HPDs of the direct heritabilities is located at the region greater than zero. Hence, the probability of obtaining an estimate equal to zero for these parameters was almost null. Posterior distributions for the direct heritabilities of growth curve parameters are shown in Figure 1. Estimates of variance components and direct heritabilities for growth curve parameters showed no or slight differences between median, mode and mean. Therefore, a normal posterior distribution was observed for direct heritabilities. Abegaz et al. [3] reported heritabilities of 0.29, 0.18 and 0.14 for A, B and K parameters in Horro sheep, respectively. Consistent with the current results, Bathaei and Leroy [19], Stobart et al. [20] and Stobart [21] reported high estimates of heritability for A parameter. On the other hand, Mavrogenis and Constantinou [22] and Näsholm [23] reported lower heritabilities for this parameter in Chios sheep and Swedish Finewool sheep, respectively. The higher heritability for mature weight is probably due to maturity at a later age in Guilan sheep, where the influence of environmental factors becomes small. In general, several factors such as breed of the animal, genetic variation within population, management and environmental conditions, the method of estimating parameter, etc., would have affected the differences between estimations [10]. The heritability and genetic relationship between traits are needed for planning an efficient breeding system and development of effective genetic evaluation. The current estimates of genetic parameters for growth curve characteristics in Guilan sheep could be used for designing future selection schemes in this breed.

**Table 1:** **The posterior mean, median and mode of direct genetic, residual and phenotypic variances and direct heritabilities for growth curve parameters of Guilan sheep [standard deviations (PSD) of marginal posterior distribution of mean estimates are in brackets]**

**Parameter**	**Item**	**Mean**	**Median**	**Mode**	**HPD**
A	${\sigma}_a^2$	32.38 (3.50)	32.40	29.85	25.77-39.51
	${\sigma}_e^2$	50.74 (3.34)	50.87	48.91	44.23-57.18
	${\sigma}_p^2$	83.13 (1.32)	83.09	83.03	80.50-85.66
	${h}_a^2$	0.39 (0.04)	0.39	0.37	0.31-0.47
B	${\sigma}_a^2$	5.86 (1.70)	5.86	4.46	2.62-8.91
	${\sigma}_e^2$	19.37 (1.66)	19.35	19.13	16.31-22.54
	${\sigma}_p^2$	25.23 (0.42)	25.24	25.09	24.47-26.05
	${h}_a^2$	0.23 (0.07)	0.23	0.24	0.10-0.35
K	${\sigma}_a^2$	0.39 (0.19)	0.36	0.19	0.11-0.78
	${\sigma}_e^2$	9.64 (0.25)	9.66	9.73	9.15-10.09
	${\sigma}_p^2$	10.03 (0.16)	10.03	9.99	9.74-10.35
	${h}_a^2$	0.039 (0.019)	0.035	0.019	0.011-0.078

*h*_*a*_^2^ = Direct heritability; *σ*_*a*_^2^ = Direct additive genetic variance; *σ*_*e*_^2^ = Residual variance; *σ*_*p*_^2^ = Phenotype variance; HPD = 95% high posterior density interval.

**Figure 2:**
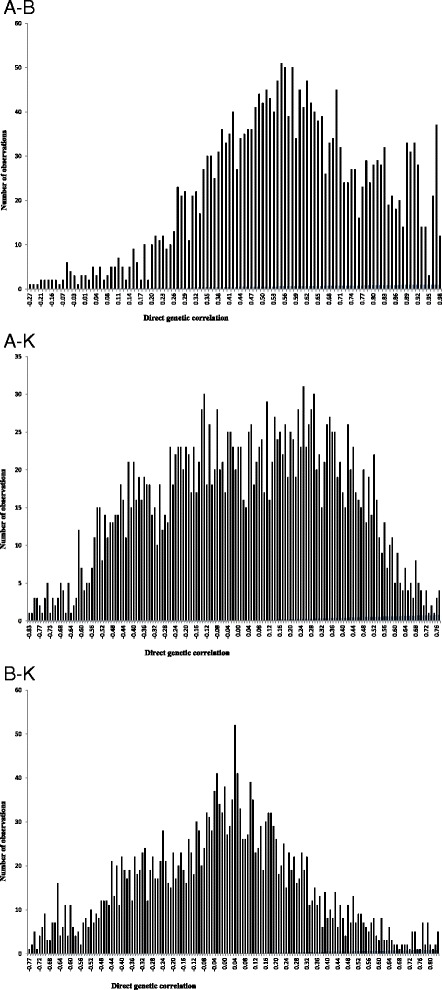
Posterior distribution of direct genetic correlations between growth curve parameters (**A**-**B**, **A**-**K** and **B**-**K**).

The posterior mean, median and mode of direct genetic, residual and phenotypic correlations between growth curve parameters of Guilan sheep are reported in Table 2. Estimates of direct genetic correlation between growth curve parameters were 0.57, 0.03 and −0.01 between A-B, A-K and B-K, respectively. The positive and high genetic correlation between A and B parameters is evident as expected for common genetic and physiological mechanisms controlling these traits. Positive genetic correlation between these traits suggests that selection in favour of one parameter of the growth curve would also improve the other parameter. On the other hand, genetic correlations between A-K and B-K were not different from zero. Therefore, changing genetically one of these parameters did not affect the other trait. This finding is in contrast with those reported by Abegaz et al. [3], Bathaei and Leroy [19] and Stobart et al. [21]. Positive genetic correlation between A-B indicated that heavy lambs at birth had greater mature weight. Therefore, medium to high heritabilities for initial and mature weights along with positive genetic correlations between these traits make it clear that genetic changes in growth patterns can be accomplished. The variation range of HPDs for the direct genetic correlations of A-K and B-K includes zero which indicates that there was a probability of obtaining zero estimates for these correlations. The HPD interval of genetic correlation between A-B did not include zero. Posterior distributions of direct genetic correlations between growth curve parameters are shown in Figure 2. There were generally no or slight differences between median, mode and mean estimates of direct genetic correlations. Therefore, a normal posterior density could be assumed for direct genetic correlations. Residual correlations between growth curve parameters varied form −0.52 (between A-K) to 0.48 (between A-B). Phenotypic correlations between growth curve parameters varied form −0.49 (between A-K) to 0.47 (between A-B). In general, the HPD interval of residual and phenotypic correlations did not include zero; therefore, these correlations were assumed to be different from zero. The negative residual correlation between A-K in this study indicated that the provision of appropriate environmental conditions for early maturity could not have positive effects on mature weight. This negative residual correlation could result in negative phenotypic correlation between these traits because corresponding genetic correlation was near to zero.

**Table 2:** **The posterior mean, median and mode of genetic, residual and phenotypic correlations between growth curve parameters of Guilan sheep [standard deviations (PSD) of marginal posterior distribution of mean estimates are in brackets]**

**Correlation**	**Item**	**Traits**		
		**A-B**	**A-K**	**B-K**
${r}_{a_1{a}_2}$	Mean	0.57 (0.22)	0.03 (0.34)	−0.01 (0.32)
	Median	0.57	0.05	0.00
	Mode	0.55	0.25	0.04
	HPD	0.20-0.98	−0.61-0.61	−0.67-0.58
${r}_{e_1{e}_2}$	Mean	0.48 (0.03)	−0.52 (0.02)	−0.32 (0.03)
	Median	0.48	−0.52	−0.32
	Mode	0.48	−0.52	−0.32
	HPD	0.42-0.53	−0.55- -0.48	−0.37- -0.27
${r}_{p_1{p}_2}$	Mean	0.47 (0.01)	−0.49 (0.01)	−0.28 (0.01)
	Median	0.47	−0.49	−0.28
	Mode	0.47	−0.49	−0.28
	HPD	0.46-0.49	−0.50- -0.47	−0.30- -0.26

${r}_{a_1{a}_2}$ = Direct genetic correlation between growth curve parameters; ${r}_{e_1{e}_2}$ = Residual correlation between growth curve parameters; ${r}_{p_1{p}_2}$ = Phenotypic correlation between growth curve parameters; HPD = Highest posterior density at 95%.

Estimates of direct genetic trends for A, B and K parameters were positive with values of 0.014 ± 0.003 (P < 0.001), 0.0012 ± 0.0009 (P > 0.05) and 0.000002 ± 0.0001 (P > 0.05), respectively. Estimates of phenotypic trends for A, B and K parameters were significantly positive (P < 0.001), the corresponding values being 0.12 ± 0.02, 0.22 ± 0.01 and 0.07 ± 0.01, respectively. There were no estimates of genetic trends for sheep growth curve parameters in literature. Accurate prediction of the breeding values of animals is one of the best tools available to maximize response to a selection plan [17]. The success of a breeding program can be assessed by testing the actual change in breeding value expressed as a proportion of expected theoretical change in the mean of breeding value for the trait under selection [24]. The positive and significant direct genetic trend for A parameter in this study indicates that the genetic merit for this parameter was increased over the years in Guilan sheep.

## Conclusions

Current genetic estimates for growth curve parameters in Guilan sheep could be applied in designing selection program in this breed. Positive and significant direct genetic trend for A growth curve parameter of Guilan sheep was obtained in the current study. A and B parameters had the highest estimates of direct heritability and a high genetic correlation, they may be improved by genetic selection and could be included in genetic improvement programs for this breed. Also, it is important to develop an optimal selection strategy to achieve a desired shape of growth curve through changing genetically the parameters of model.
